# Effectiveness and sustainability of a motor-cognitive stepping exergame training on stepping performance in older adults: a randomized controlled trial

**DOI:** 10.1186/s11556-020-00248-4

**Published:** 2020-09-29

**Authors:** Klaus Hauer, Elena Litz, Michaela Günther-Lange, Caroline Ball, Eling D. de Bruin, Christian Werner

**Affiliations:** 1 AGAPLESION Bethanien Hospital, Geriatric Center of Heidelberg University, Heidelberg, Germany; 2 Institute of Human Movement Sciences and Sport, Department of Health Sciences and Technology, ETH Zürich, HCP H 25.1, Leopold-Ruzicka-Weg 4, 8093 Zurich, Switzerland; 3 Division of Physiotherapy, Department of Neurobiology, Care Sciences and Society, Karolinska Institutet, Huddinge, Sweden; 4 Center of Geriatric Medicine, Heidelberg University, Heidelberg, Germany

**Keywords:** Older adults, Exergame, Balance, Volitional stepping, Fall prevention

## Abstract

**Background:**

Training effects reported for stepping exergames on stepping performances in older adults often based on not comprehensively validated outcomes measures, and follow-up data on their sustainability are lacking. The aim of this study is to evaluate the effectiveness and sustainability of a motor-cognitive stepping exergame training on the stepping performance in older adults.

**Methods:**

Fifty-eight older adults (78.3 ± 6.5 years) participated in the randomized controlled trial with a 10-week intervention and 10-week follow-up period. The intervention group (IG: *n* = 29) took part in a once-weekly exercise program including strength and balance exercises supplemented with an additional stepping exergame training. The control group (CG: *n* = 29) only performed the strength and balance exercises. Outcome measures included stepping reaction times (SRTs) and games scores for individual stepping exergame levels and for the overall exergame performance, as measured by an assessment strategy previously validated in older adults.

**Results:**

SRTs and/or games scores for 7 out of 10 levels and the overall exergame performance significantly improved in the IG compared to the CG during the intervention (*p* ≤ 0.001–0.039, *η*_*p*_^*2*^ = 0.090–0.445). Training gains were sustained for 2 levels and for the overall exergame performance (*p* = 0.017–0.033, *η*_*p*_^*2*^ = 0.127–0.193).

**Conclusion:**

The study demonstrates that the additional stepping exergame training effectively and sustainably improves the performance in complex motor-cognitive stepping exergame tasks in older adults, which can be relevant for preventing falls. Future research is needed to evaluate the effectiveness of such training on reducing the number of falls.

**Trial registration:**

ISRCTN registry, ISRCTN14855620, 06/06/2019 (retrospectively registered).

## Introduction

The ability to initiate a fast voluntary step or to inhibit a preplanned step and adjust the foot landing position to prevent loss of balance or obstacle contact is crucial for avoiding a fall [1–3]. However, this ability deteriorates with age [4–6] and poor reactive and volitional stepping, especially impaired response inhibition during stepping, have been identified as risk factors for falls in older adults [7–12]. Exercise interventions with focus on performing precise, rapid and well-coordinated steps thus represent a valuable approach in fall prevention. In a systematic review, it has been shown that reactive and volitional stepping exercises can reduce falls in older adults by approximately 50% [13]. Improvements in balance and reaction time as well as the high task specificity for dynamic postural control due to the involvement of associated sensory, cognitive and motor skills were suggested to contribute to the high fall reduction effects of stepping exercises [13].

Interactive, exercise-based video gaming (exergaming) can offer attractive and effective training tools to improve stepping ability in older adults [14–16]. Stepping exergames typically require players to perform volitional and inhibitory stepping movements while interacting with stepping targets and distractors [14, 15], or trigger reactive stepping when balance is disturbed under unstable surface conditions [17] to complete computer-controlled, cognitively challenging tasks projected onto a display screen. Such complex motor-cognitive stepping exergame tasks involve the interaction between the sensory, information-processing (e.g. dual tasking, inhibiting irrelevant stimuli, fast decision-making) and neuromuscular systems during controlled body weight transfers. This interaction between multiple functional systems is similar to that required to initiate step responses to prevent falls [18], which seems more difficult to reproduce through traditional stepping exercises. Further advantages of exergame-based stepping exercises compared to traditional stepping exercises involve the opportunity to track players’ performance during interactive gameplay on various specific parameters by means of internal-software-generated data, which can be used not only to provide real-time immediate performance feedback but also to adjust the task difficulty to the specific performance level of the individual participant or be used for highly-specific assessment of the multimodal, motor-cognitive task characteristics of the specific exergame [19–22]. Furthermore, the playful and entertaining character of interactive exergaming can make training routine more engaging and enjoyable therefore increasing motivation to exercise [23] shifting participants’ attention to positive experience of movement [18], which might help to encourage older people to participate in physical activity [24] and to enhance the low participation and intervention adherence observed for conventional exercise programs [25].

Previous randomized controlled trials (RCTs) have shown that stepping exergame interventions can be effective to improve stepping performance in older adults, thereby reducing their fall risk [14–16, 26]. In these RCTs, intervention effects were partly documented by task-specific outcome measures on stepping performance derived from the internal data flow of the stepping exergame used for training purposes [14, 15]. However, the ‘internal outcome measures” applied in these RCTs had not previously been comprehensively validated for assessment purposes. In addition, there is a general lack of RCTs that examine the sustainability of stepping exergame interventions on the stepping performance of older adults after a long-term follow-up period without exergame training.

A previous RCT evaluating the effectiveness of a 10-week, non-stepping exergame-based balance training program (2 × 10 min/week) in older persons with cognitive impairment (CI) using validated internal outcome measures identified sustainable task-specific intervention effects up to three months after training cessation [20] and a curvilinear dose-response relationship, with most training gains allocated to the initial training phase and effects leveling off for further training sessions [27].

In summary, the aim of this study was to assess the effects of a 10-week task-specific motor-cognitive stepping exergame training on stepping performance using comprehensively validated internal outcome measures and to investigate the sustainability of training gains in older adults. Based on previous findings on the dose-response relationship and the sustainability of non-stepping exergame-based balance training in older persons with CI [20, 27], we hypothesized that significant and sustainable task-specific improvements in stepping performance can be achieved with a restricted training frequency and duration (1 × 20 min/week) in older persons without CI.

## Methods

### Study design

The study was designed as a single-blinded (assessor) RCT with a 10-week intervention and 10-week follow-up period (trial registration: ISRCTN14855620). The study protocol was approved by the Ethics Committee of the Medical Faculty of Heidelberg in accordance with the Helsinki Declaration (protocol number: S-242/2015).

### Study population

Participants were recruited through direct contact from an ambulant geriatric rehabilitation sports club for older persons with limited functional status (REGE e.V.), which is associated to a German geriatric hospital. Inclusion criteria were as follows: 60 years or older [28]; no CI (Mini-Mental State Examination [MMSE] score ≥ 24) [29]; ability to walk independently with or without walking aid; no uncontrolled or terminal neurologic, cardiovascular, metabolic or psychiatric disorder, and written informed consent. Eligible participants were randomly assigned, stratified by sex, to the intervention (IG) or control group (CG) using an urn design for clinical trials [30]. A person unrelated to the study performed the randomization procedure.

A power analysis conducted with G*Power version 3.1.9.2 [31] for a repeated-measures design with two measurements, two groups, a correlation between repeated measures of 0.5, a two-sided significance level (α) of 0.05 and a power (1-β) of 0.95 revealed that a sample size of 54 participants is required to detect a moderate effect (*η*_*p*_^*2*^ > .0588). Considering potential drop-outs during the intervention period, the sample size was set to 58 participants. The assumption of moderate effects was based on a comparable RCT on the effectiveness of a exergame-based balance training in older persons with CI [20].

### Intervention

Both the IG and CG continued to engage in their weekly strength and balance exercises in the ambulation rehabilitation sports club over the entire 20-week study period, with one session à 90 min per week.

**Fig. 1:**
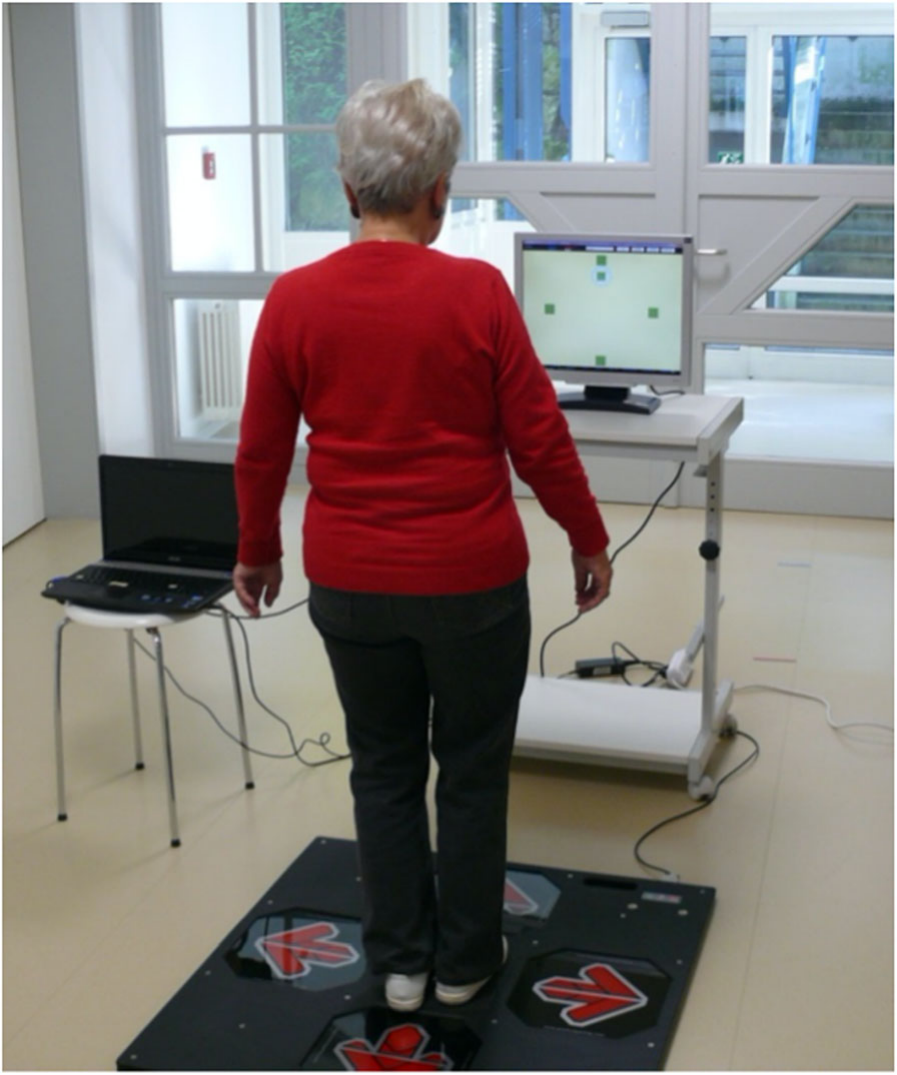
Participant performing stepping exergame tasks on the pressure sensitive platform

**Fig. 2:**
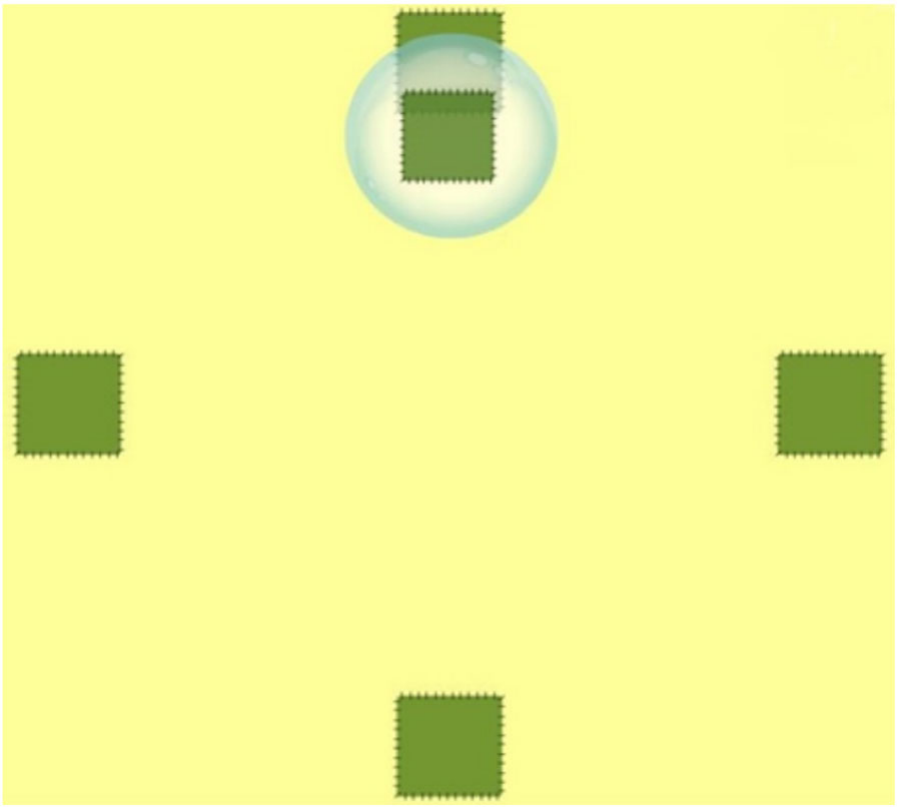
Stepping exergame task cueing a forward step. (from Litz et al. *Games Health J* 2019 [19], with permission of Mary Ann Liebert, Inc. Copyright© 2019 Mary Ann Liebert, Inc.)

Participants allocated to the IG additionally received a 10-session individual supervised exergame-based stepping training during the intervention period for 20 min each training session. The stepping exergame was performed on an interactive training device (Impact Dance Platform, Positive Gaming BV, BZ Haarlem, Netherlands) – a pressure sensitive plate (87.5 × 87.5 × 2.5 cm) running with a game software developed by Dividat® (Dividat AG, Schindellegi, Switzerland) and connected to a laptop and a monitor by a USB port (Fig. 1). Participants’ step position and step timing were tracked by contact sensors located within pressure sensitive areas marked with an arrow corresponding to the direction of the step (forwards, backwards, right, left). To play the game, participants were standing on the central section of the platform, feet closely parallel. The arrangement of squares on the platform was reproduced on the screen. Steps were cued by squares randomly moving from the central position to one of the four static squares (Fig. 2). When executing the indicated steps, participants had to meet the point in time when a moving square was superimposed on a static one. Before conducting the next step, participants had to return to the central position. Visual feedback was provided real-time after each step. The game included 10 levels à 60 s, split in two sub-sessions. In sub-session 1, progressive difficulty was achieved by increasing the task rate, as well as by additional step directions involved in the level (Table 1). Sub-session 2 additionally included randomly-presented tasks targeting inhibitory control: Participants had to restrain reaction when instead of a moving square a triangle appeared (levels 6–9), and to react also to moving triangles if static squares switched to triangles (level 10). During each exergame training session, participants trained the different levels of the sub-session in a standardized sequence, starting with the lowest level and moving to higher levels. The decision point for moving to a higher level in both sub-sessions was set at a minimum of 50% of successfully executed actions (steps) conducted in the indicated direction within 320 ms, or successful inhibitions within a level. All participants were instructed and supervised by a medical student who had been adequately trained in the training procedure. Training instructions were standardized across all participants and training sessions. During the training sessions, no physical or verbal assistance was allowed. Game performance was documented at each training session.

**Fig. 3:**
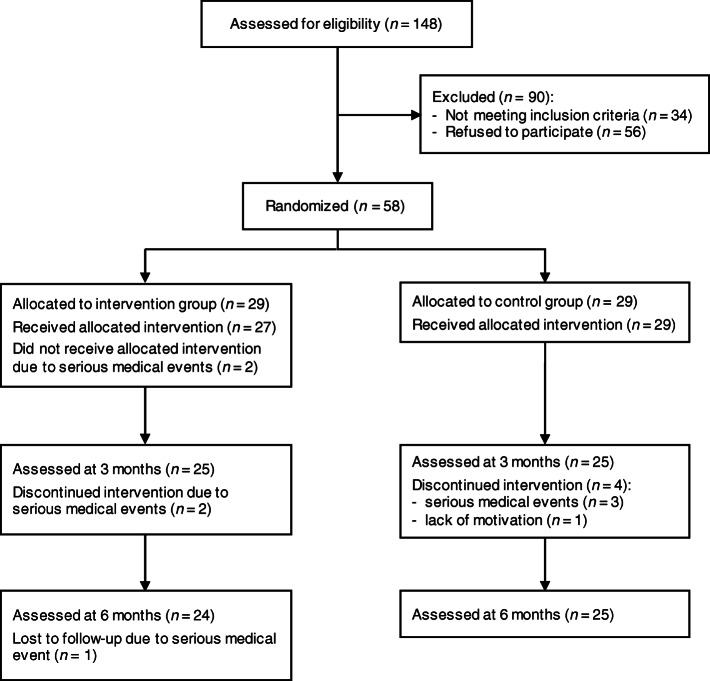
Flow chart for screening, recruitment, allocation, intervention, follow-up, and data analysis

**Table 1:** Task rates, stepping directions and inhibition tasks of the different stepping exergame levels

Level	Task rate (tasks/min)	Step direction^a^	Inhibition^b^
*Sub-session 1*			
1	17	↑	–
2	17	←↑→	–
3	20	←↑↓→	–
4	26	←↑↓→	–
5	40	←↑↓→	–
*Sub-session 2*			
6	17	↑	✓
7	17	←↑→	✓
8	20	←↑↓→	✓
9	26	←↑↓→	✓
10	40	←↑↓→	✓✓

^a^ arrows indicate respective step directions involved in the level (forwards, backwards, right, left); ^b^ - levels without inhibition tasks; ✓ levels with inhibition tasks; ✓✓ level containing inhibition task with additional cognitive load

### Descriptive measures

Demographic and clinical characteristics, including age, gender, years of education, and falls in the previous 6 months were obtained by standardized interview. Body-Mass-Index (BMI) and numbers of medications were documented from medical records. Cognitive status was assessed by the MMSE [29], fear of falling by the short version of the Falls Efficacy Scale International (FES-I) [32], and health-related quality of life by the 12-item Short Form Health Survey (SF-12) [33]. Functional performance was measured by the Short Physical Performance Battery (SPPB) [34], with a SPPB score of ≤10 pt. indicating moderate functional limitations [35, 36].

### Exergame-based outcome measures

Exergame measurements were conducted before randomization (T1), at the end of the 10-week training period (T2) and at a 10-week follow-up (T3) by a blinded assessor trained in the test procedures. Identical stepping exergame tasks were used for training and assessment purposes. The assessment strategy specifically tailored to the specific training tasks included outcome measures directly derived from the internal game-based data stream, which has previously successfully and comprehensively validated in older adults, showing good feasibility, test-retest reliability and construct validity, as well as excellent sensitivity to change [19]. Primary outcome measures were defined as stepping reaction time (SRT) and game scores. SRT was specified as a deviation of step execution time from the exact superimposition of the squares or triangles on the target area and was measured in milliseconds. For each level the SRT was documented as a mean of all SRT values for this level. Depending on SRT, each stepping action was scored ranging from 0 to 5 points (SRT < 40 ms = 5 pt.; 40–80 ms = 4 pt.; 81–160 ms = 3 pt.; 161–240 ms = 2 pt.; 241–320 ms =1 pt.; > 320 ms = 0 pt), or for a step conducted earlier or in other direction than indicated in the task. Inhibition tasks were scored 1 or 0. Similar to the training procedure, exergame measurements started in both sub-sessions with the lowest level and progressed to higher levels when the participant successfully completed a level. The completion criterion for each level was the same as the decision point for moving to a higher level during the training procedure as described above (≥50% successful actions). Level adjustment ensured that the task difficulty matched participants’ individual performance. To provide the sample with an adequate overall challenge level, the stepping exergame was pretested in a pilot before the intervention. At the beginning of each exergame measurement, participants completed a practice sequence to ensure comprehension of the exergame tasks. Assessment instructions were strictly standardized across all participants.

Training adherence was documented as percentage of participants completing > 75% of sessions during the intervention period (attending ≥8 sessions out of a planned 10-session training [25].

### Statistical analysis

Descriptive data were presented as frequencies and percentages for categorical variables, and means and standard deviations (SD) or medians and interquartile ranges (IQR) for continuous variables. SRT data was log-transformed to satisfy the normality assumption for statistical analysis. For each game level, a mean SRT and a game score in percent (calculated as the participants’ level score divided by the highest possible score in this level) were computed. In addition, the mean SRTs and game scores for sub-session 1, sub-session 2 and all games levels were averaged to yield (sub-)total scores for the overall stepping performance. Taking into account the individual changes in the stepping performance of each participant over time, these (sub-)total scores were calculated for all measurement time points based on the level achieved at baseline. Statistical analyses were performed using IBM SPSS version 24.0 for Windows (IBM Corporation, Armonk, NY, USA). Unpaired t-tests, Mann-Whitney-U-tests and chi-square-tests were used as appropriate for between-group comparisons in baseline participant characteristics and training adherence. Analyses of covariance (ANCOVAs) for each game level and (sub-)total score with baseline value as covariate were used to test for significant differences between the IG and CG in absolute changes in outcome measures over the intervention period (T1-T2) and the total observation period (T1-T3). ANCOVAs for the separate observation periods were performed using an available-case approach including all participants with available data after the intervention (T2) and at the follow-up (T3), respectively (main analyses). In addition, supplementary intention-to-treat analyses for each game level and (sub-)total score were implemented, with multiple imputation (fully conditional specification [chained equations] [37], five imputations, ten iterations) to account for missing values in primary outcome measures over time. Multiple imputation models included all baseline participant characteristics (age, gender, education, falls, BMI, medications, MMSE, FES-I, SF-12, SPPB), group allocation, training adherence, and relevant outcome measures. Parameter estimates obtained from multiple imputation were either presented as pooled data as provided by SPSS or averaged over the five imputations to obtain individual estimates. Effect sizes were given as partial eta squared (*η*_*p*_^*2*^*)* and interpreted using Cohen’s thresholds: *η*_*p*_^*2*^ > .0099 (small effect), *η*_*p*_^*2*^ > .0588 (moderate effect) and *η*_*p*_^*2*^ > .1379 (large effect) [38]. Statistical significance was set at *p* < 0.05.

## Results

### Recruitment and adherence

Detailed information on screening, enrollment and drop-outs is given in Fig. 3. Members of the ambulant geriatric rehabilitation sports club (*n* = 148) were screened for eligibility, with 58 being subsequently enrolled and randomly assigned to the IG (*n* = 29) or CG (*n* = 29). Nine participants (15.5%) dropped out (IG: *n* = 5 [17.2%], CG: *n* = 4 [13.8%]; *p* = 0.717). Drop-outs were related to serious medical events (*n* = 6) and lack of motivation (n = 1). No falls or other critical events occurred during the stepping exergame training, and all serious medical events were not directly or indirectly attributable to the stepping exergame. Comparisons of participants who stayed in the study with those who dropped out revealed no significant differences for descriptive variables at baseline in both study groups (IG: *p* = 0.065–0.880; CG: *p* = 0.055–0.964). During the intervention period, 62.1% of participants in the IG and 75.0% of participants in the CG attended ≥8 out of 10 planned training sessions, with no significant between-group differences (*p* = 0.294).

### Participant characteristics

Fifty-eight community-dwelling, cognitively intact older adults with on average moderate functional limitations and relevant comorbidities participated in the study (Table 2). Except for the MMSE, no significant differences at baseline between the IG and the CG were found for descriptive variables. The one-point between-group discrepancy in the MMSE score does not represent a clinically relevant mean difference [39], with all participants having no CI as defined by the inclusion criterion of a MMSE score ≥ 24.

**Table 2:** Participant characteristics

Characteristics	IG (***n*** = 29)	CG (***n*** = 29)	***P***-value
Age, years	79.0 ± 5.9	77.6 ± 7.1	0.412^a^
Gender, females	27 (93.1)	27 (93.1)	> 0.999^b^
Education, years	12.5 [11.0–15.0]	13.0 [10.0–15.0]	0.778^c^
Mini Mental State Examination, score	27.5 ± 1.8	28.5 ± 1.4	0.017 ^a^
Body-Mass-Index, kg/m^2^	27.5 ± 5.2	26.2 ± 5.0	0.360 ^a^
Medication, n	4.8 ± 2.9	5.2 ± 4.1	0.711 ^a^
Falls Efficacy Scale-International, score	8.6 ± 2.4	8.5 ± 2.3	0.886 ^a^
Recent history of falls, *n*	2 (6.9)	6 (21.4)	0.114^b^
Short Physical Performance Battery, score	9.45 ± 2.41	10.31 ± 1.76	0.127^a^
12-item Short Form Health Survey, score			
Physical component	39.8 ± 2.2	41.0 ± 1.6	0.065^a^
Mental component	53.2 ± 1.5	54.8 ± 1.4	0.486^a^

Data given as mean ± SD, frequency (%), or median [IQR]. *IG* intervention group, *CG* control group, *n* = number of participants, *p*-values given for ^a^t-tests, ^b^chi-square and ^c^Mann-Whitney-U-tests to test for differences between IG and CG; ^d^ based on *n* = 57 due to missing data for one participant.

### Effects of the intervention

Significant improvements in the IG compared to the CG were observed for most SRT outcomes over the intervention period (available-case analysis; Table 3). As an effect of the stepping exergame training, the SRTs significantly decreased in almost all levels without inhibition tasks (sub-session 1, level 1–4: *p* ≤ 0.001–0.036, *η*_*p*_^*2*^ = 0.120–468), except for the most complex level (*p* = 0.955, *η*_*p*_^*2*^ < 0.001). In the levels with inhibition tasks (sub-session 2), significant between-group changes over the intervention period in favor of the IG were only found in the initial level 6 (*p* = 0.039, *η*_*p*_^*2*^ = 0.090) and the most complex level 10, (*p* = 0.001, *η*_*p*_^*2*^ = 0.341). All (sub-)total SRT scores significantly improved in the IG compared to the CG, with overall large effect sizes (*p* ≤ 0.001–0.003, *η*_*p*_^*2*^ = 0.341–0.445).

**Table 3:** Effects of the stepping exergame training on stepping reaction time [ms] (available-case analysis)

	T1		T2		T3		T1-T2			T1-T3		
	*n*	Median (IQR)	*n*	Median (IQR)	*n*	Median (IQR)	% change* Mean ± SD	*P*-value^†^	*η*_*p*_^*2*†^	% change* Mean ± SD	*P*-value^†^	*η*_*p*_^*2*†^
*Sub-session 1*												
Level 1												
CG	29	150 (116–222)	24	127 (94–213)	25	118 (88–150)	−2.4 ± 40.8	**< 0.001**	0.237	−21.9 ± 37.9	0.587	0.006
IG	29	200 (153–241)	25	95 (71–134)	24	111 (89–144)	−44.3 ± 20.6			−33.8 ± 29.9		
Level 2												
CG	24	178 (130–246)	23	170 (132–209)	25	110 (85–184)	−7.4 ± 26.2	**0.001**	0.235	−25.6 ± 35.4	0.667	0.005
IG	24	273 (222–364)	24	123 (96–173)	24	131 (91–169)	−48.7 ± 31.6			−47.8 ± 28.0		
Level 3												
CG	22	151 (119–210)	22	154 (122–207)	24	134 (89–183)	+ 8.8 ± 42.3	**0.036**	0.120	−16.0 ± 28.0	**0.033**	0.127
IG	21	222 (194–285)	22	102 (80–178)	23	135 (81–149)	−44.7 ± 33.9			−49.3 ± 24.4		
Level 4												
CG	19	125 (112–189)	23	133 (117–181)	23	144 (99–195)	+ 7.4 ± 49.7	**0.030**	0.147	+ 9.7 ± 45.1	**0.017**	0.193
IG	17	148 (132–197)	20	100 (85–145)	20	119. (87–138)	−31.8 ± 18.3			−28.1 ± 17.8		
Level 5												
CG	14	169 (117–209)	12	152 (110–173)	17	132 (108–217)	+ 0.8 ± 45.9	0.955	< 0.001	+ 5.4 ± 37.4	0.861	0.002
IG	7	159 (86–211)	15	120 (79–183)	14	114 (81–150)	+ 13.0 ± 87.3			+ 20.8 ± 72.9		
Sub-total score												
CG	29	175 (142–218)	24	157 (129–191)	25	141 (104–177)	−6.9 ± 20.3	**< 0.001**	0.468	− 22.1 ± 23.7	0.056	0.077
IG	29	222 (177–254)	25	118 (85–152)	24	126 (95–166)	−46.2 ± 18.6			− 39.9 ± 19.6		
*Sub-session 2*												
Level 6												
CG	29	159 (108–223)	25	132 (101–179)	24	138 (90–163)	+ 10.7 ± 74.5	**0.039**	0.090	−5.9 ± 58.1	0.115	0.054
IG	29	156 (109–248)	24	103 (83–159)	24	91 (74–154)	−24.4 ± 35.6			−31.3 ± 30.0		
Level 7												
CG	29	147 (116–246)	25	108 (92–157)	24	137 (80–177)	−9.1 ± 51.0	0.095	0.058	−4.5 ± 51.9	0.169	0.042
IG	29	164 (104–252)	25	81 (69–142)	24	114 (80–151)	−31.4 ± 40.0			−24.9 ± 43.0		
Level 8												
CG	28	136 (98–223)	24	111 (93–142)	24	128 (91–194)	−9.7 ± 50.5	0.415	0.015	+ 13.6 ± 74.6	0.152	0.046
IG	29	152 (111–211)	25	101 (81–163)	24	105 (82–148)	−26.3 ± 25.0			−15.4 ± 61.0		
Level 9												
CG	28	133 (105–191)	24	130 (86–190)	23	116 (90–143)	+ 5.9 ± 51.1	0.092	0.060	−3.8 ± 52.9	0.551	0.008
IG	29	171 (112–216)	25	143 (86–177)	24	123 (76–184)	−18.8 ± 37.8			−16.9 ± 46.6		
Level 10												
CG	22	213 (135–283)	17	208 (138–298)	19	152 (124–322)	+ 6.7 ± 24.3	**0.001**	0.341	−10.7 ± 39.4	0.707	0.005
IG	21	292 (215–403)	20	200 (97–257)	19	189 (133–283)	−44.2 ± 30.7			−34.5 ± 33.8		
Sub-total score												
CG	29	169 (129–213)	25	129 (113–182)	24	135 (98–161)	−4.4 ± 46.4	**0.003**	0.173	−13.1 ± 26.3	**0.026**	0.106
IG	29	198 (137–261)	25	128 (84–157)	24	128 (89–165)	−34.5 ± 20.2			−31.5 ± 23.8		
*Total score (sub-session 1 & 2)*												
CG	29	177 (144–210)	25	145 (121–178)	25	136 (109–181)	−5.7 ± 28.2	**< 0.001**	0.445	−18.2 ± 21.8	**0.012**	0.131
IG	29	204 (164–263)	25	128 (84–156)	24	127 (91–166)	−42.4 ± 12.9			−37.6 ± 17.1		

Stepping reaction times were log-transformed to satisfy the normality assumption for statistical analysis

* calculated as follows: ((retest score – baseline score) / baseline score) × 100

^†^ *P*-values and effect sizes (*η*_*p*_^*2*^*)* are given for group effects with adjustment for baseline covariates as calculated by analysis of covariance (ANCOVA). Significant *p*-values < 0.05 are marked in bold

*T1* baseline assessment before training, *T2* assessment after the 10-week training period, *T3* assessment 10 weeks after training cessation, *IQR* interquartile range, *SD* standard deviation, *CG* control group, *IG* intervention group

Significant improvements in favor of the IG compared to the CG were also obtained for most game score outcomes (available-case analysis; Table 4). Games scores significantly increased in the first three levels of sub-session 1 (level 1–3: *p* = 0.001–0.006, *η*_*p*_^*2*^ = 0.141–0.232). In the levels of sub-session 2, significant improvements in games scores were observed for level 7 (*p* = 0.005, *η*_*p*_^*2*^ = 0.154) and the most complex level 10 (*p* = 0.011, *η*_*p*_^*2*^ = 0.185). All (sub-)total game scores significantly improved as an effect of the stepping exergame, with overall large effect sizes (*p* ≤ 0.001, *η*_*p*_^*2*^ = 0.185–0.364).

**Table 4:** Effects of the stepping exergame training on game scores [%] (available-case analysis)

	T1		T2		T3		T1-T2			T1-T3		
	*n*	Mean ± SD	*n*	Mean ± SD	*n*	Mean ± SD	% change* Mean ± SD	*P*-value^†^	*η*_*p*_^*2*†^	% change* Mean ± SD	*P*-value^†^	*η*_*p*_^*2*†^
*Sub-session 1*												
Level 1												
CG	29	54.9 ± 13.5	24	56.3 ± 16.3	25	62.6 ± 14.4	+ 9.0 ± 46.4	**0.009**	0.141	+ 21.1 ± 32.8	0.476	0.011
IG	29	45.9 ± 12.2	25	66.6 ± 13.1	24	62.6 ± 13.6	+ 53.3 ± 52.2			+ 42.0 ± 46.6		
Level 2												
CG	24	47.9 ± 12.9	23	49.0 ± 13.9	25	58.0 ± 15.4	+ 8.0 ± 33.8	**0.001**	0.232	+ 32.3 ± 45.9	0.556	0.009
IG	24	39.0 ± 11.7	24	61.3 ± 14.6	24	59.1 ± 14.2	+ 74.5 ± 59.2			+ 64.6 ± 56.2		
Level 3												
CG	22	51.6 ± 10.8	22	50.1 ± 11.6	24	56.7 ± 12.5	−0.5 ± 22.5	**0.006**	0.193	+ 12.7 ± 23.4	0.088	0.083
IG	21	40.0 ± 9.6	22	58.1 ± 13.3	23	58.0 ± 13.6	+ 56.5 ± 46.2			+ 60.1 ± 48.4		
Level 4												
CG	20	59.0 ± 13.5	23	55.0 ± 12.9	23	57.7 ± 13.5	+ 4.1 ± 35.3	0.333	0.030	+ 4.9 ± 37.7	0.104	0.092
IG	17	52.3 ± 8.8	20	61.4 ± 14.5	20	62.0 ± 14.4	+ 16.0 ± 19.4			+ 19.1 ± 17.9		
Level 5												
CG	14	47.2 ± 14.9	12	48.6 ± 12.9	17	48.7 ± 19.0	+ 9.8 ± 40.5	0.995	0.001	−0.9 ± 33.5	0.914	0.001
IG	7	52.0 ± 18.1	15	56.5 ± 19.0	14	55.1 ± 19.2	+ 7.8 ± 55.3			+ 6.0 ± 62.3		
Sub-total score												
CG	29	50.8 ± 9.9	24	51.6 ± 10.2	25	56.5 ± 10.8	+ 3.5 ± 21.7	**< 0.001**	0.330	+ 15.4 ± 21.7	0.063	0.073
IG	29	44.1 ± 10.0	25	61.6 ± 12.2	24	58.7 ± 12.1	+ 44.2 ± 30.2			+ 34.2 ± 29.2		
*Sub-session 2*												
Level 6												
CG	29	52.7 ± 18.5	25	55.4 ± 15.7	24	58.5 ± 16.8	+ 16.9 ± 56.9	0.133	0.047	+ 25.6 ± 70.0	0.111	0.055
IG	29	51.3 ± 17.3	24	61.9 ± 18.6	24	65.3 ± 14.4	+ 30.3 ± 51.1			+ 34.2 ± 52.8		
Level 7												
CG	29	56.7 ± 13.0	25	60.2 ± 14.8	24	60.7 ± 17.2	+ 6.7 ± 28.2	**0.005**	0.154	+ 5.7 ± 35.4	0.126	0.051
IG	29	51.0 ± 19.4	25	67.3 ± 14.8	24	63.6 ± 16.4	+ 67.8 ± 129.2			+ 60.7 ± 149.8		
Level 8												
CG	28	52.4 ± 16.0	24	59.0 ± 13.8	24	52.8 ± 17.7	+ 17.4 ± 36.1	0.246	0.029	+ 5.8 ± 39.2	0.070	0.073
IG	29	49.5 ± 14.9	25	60.0 ± 15.9	24	59.7 ± 16.8	+ 26.3 ± 33.3			+ 27.9 ± 50.3		
Level 9												
CG	28	57.1 ± 13.7	24	55.8 ± 14.0	23	59.5 ± 16.0	−2.8 ± 26.7	0.128	0.050	+ 2.7 ± 30.9	0.454	0.013
IG	29	50.9 ± 17.8	25	57.2 ± 20.8	24	59.0 ± 19.8	+ 28.6 ± 61.2			+ 37.8 ± 99.4		
Level 10												
CG	22	36.8 ± 19.5	20	39.9 ± 19.6	20	43.6 ± 19.8	+ 9.1 ± 26.7	**0.011**	0.185	+ 35.7 ± 26.7	0.423	0.022
IG	21	27.7 ± 19.2	21	40.8 ± 25.5	20	41.6 ± 22.3	+ 168.6 ± 218.6			+ 183.8 ± 254.1		
Sub-total score												
CG	29	49.8 ± 12.4	25	54.2 ± 12.2	24	55.3 ± 12.7	+ 6.7 ± 24.6	**0.002**	0.185	+ 6.9 ± 22.0	**0.016**	0.122
IG	29	44.1 ± 13.9	25	58.8 ± 15.1	24	56.5 ± 14.5	+ 43.2 ± 39.8			+ 36.8 ± 41.2		
*Total score (sub-session 1 & 2)*												
CG	29	49.3 ± 10.4	24	52.2 ± 10.2	25	55.2 ± 11.3	+ 4.1 ± 17.2	**< 0.001**	0.364	+ 10.8 ± 17.1	**0.008**	0.142
IG	29	43.6 ± 10.9	25	59.5 ± 13.1	24	57.3 ± 13.2	+ 42.3 ± 30.4			+ 33.7 ± 28.3		

* calculated as follows: ((retest score – baseline score) / baseline score) × 100

^†^ *P*-values and effect sizes (*η*_*p*_^*2*^*)* are given for group effects with adjustment for baseline covariates as calculated by analysis of covariance (ANCOVA). Significant *p*-values < 0.05 are marked in bold

*T1* baseline assessment before training, *T2* assessment after the 10-week training period, *T3* assessment 10 weeks after training cessation, *SD* standard deviation, *CG* control group, *IG* intervention group

Results of the intention-to-treat analysis were comparable to those found in the available-case analysis. Significant improvements in the IG compared to the CG were observed for SRTs and/or game scores in levels 1, 2, 4, 6, 7 and 10 (*p* = 0.003–0.047, *η*_*p*_^*2*^ = 0.070–0.208) and for all (sub-)total scores (*p* ≤ 0.001–0.028, *η*_*p*_^*2*^ = 0.088.-0.208). Additional files 1 and 2 provided the detailed results of the intention-to-treat analysis.

Completion rates were high for the lowest levels of the sub-sessions (level 1 and 6 = 100%), but expectedly decreased throughout the game levels with progression to more advanced sub-session levels, leading to subsample variation between distinct game levels (Tables 3 and 4). The completion rate in sub-session 1 decreased to a higher extent with increasing level than that in sub-session 2.

### Sustainability of effects

Training-induced improvements in SRT and game score outcomes were sustained 10-weeks after training cessation for individual levels, for total sub-sessions, and for the total session (available-case analysis; Tables 3 and 4).

Significant sustained effects of the stepping exergame training on the SRT at follow-up were observed for the levels 3 (*p* = 0.033, *η*_*p*_^*2*^ = 0.127) and level 4 (*p* = 0.017, *η*_*p*_^*2*^ = 0.193) without inhibition tasks (sub-session 1). No significant between-group changes were found in the levels 6–10 (sub-session 2) with inhibition tasks (*p* = 0.115–0.707, *η*_*p*_^*2*^ = 0.005–0.054). All (sub-)total SRT scores were still significantly improved or tended to be improved at follow-up in the IG as compared to the CG, with moderate effect sizes (*p* = 0.012–0.056, *η*_*p*_^*2*^ = 0.077–0.131).

Game scores without inhibition tasks showed a trend to sustained moderate effects of the stepping exergame training for the levels 3 and 4 as well as for the total sub-session but missed the level of significance (*p* = 0.063–0.104, *η*_*p*_^*2*^ = 0.073–0.092). A trend to sustained moderate effects were also observed for the game scores in level 7 (*p* = 0.126, *η*_*p*_^*2*^ = 0.051) and level 8 (*p* = 0.070, *η*_*p*_^*2*^ = 0.073). The (sub-)total game scores for sub-session 2 (*p* = 0.016, *η*_*p*_^*2*^ = 0.122) and the total session (*p* = 0.008, *η*_*p*_^*2*^ = 0.142) showed significantly sustained training gains in the IG compared to the CG, with moderate to large effect sizes.

Results of the intention-to-treat analysis were comparable to those found in the available-case analysis (see Additional files 1 and 2). Significant sustained improvements in the IG compared to the CG were observed for the SRTs in level 3 and 4 (*p* = 0.012–0.056, *η*_*p*_^*2*^ = 0.077–0.131), and all (sub-)total SRT and games scores were also still significantly improved or showed a trend to be still improved at follow-up (*p* = 0.019–.0.118, *η*_*p*_^*2*^ = 0.044–0.202).

## Discussion

The study demonstrates that the added stepping exergame training effectively improves performance in complex motor-cognitive stepping exergame tasks in older adults. Training-induced gains were maintained 10 weeks after training cessation, indicating the long-term effectiveness of the stepping exergame training to sustainably improve exergame-based stepping performances which may be highly relevant for avoiding falls. To the best of our knowledge, this is the first study to provide data on the effectiveness and sustainability of a stepping exergame training in improving complex motor-cognitive stepping performances assessed by comprehensively validated internal outcome measures [19].

The design of the present study stands out with a rather short training period (10 weeks), a limited duration of the single training sessions (1 × 20 min/week) and the fact that the training was embedded in an effective strength and functional training with a control group performing an identical training except for the specific stepping exergame training to be evaluated. The present results therefore differ from other studies with focus on mere effectiveness of training programs testing single effects. However, the potential use case of instrumented (and therefore expensive) exergame training would be group training sessions in medical rehabilitation settings, sports clubs, or commercial locations. Exergame training in such settings would mainly be used in addition to other forms of training with a specific additional focus on motor-cognitive training and as a motivational approach attractive for persons which may otherwise not take part in established training activities. With the present study design we mimic such a real-life scenario, testing effects in addition to established training programs. However, this realistic scenario is dealt for a control group representing a high challenge setting high stakes with respect to achieve significant between group differences for any additional training effect that require a high specificity of tasks as in a demanding motor- cognitive training via stepping exergame tasks not represented in established training programs.

### Effects of the intervention

After undertaking the stepping exergame training, older people substantially improved their performance in stepping exergame tasks targeting the interaction between multiple domains of sensory, cognitive and motor functioning highly relevant for maintaining dynamic postural control and avoiding falls [18]. The complex motor-cognitive tasks used in the stepping exergame combine volitional stepping training as one of the most effective strategies in fall prevention [13, 40] with cognitive demands associated with fall risk, such as reaction time, perceptual speed and visuospatial attention, and additional response inhibition components [41–43]. The substantial improvements in these complex motor-cognitive tasks, as documented by large effect sizes for SRT and game scores, were observed after a relatively short training period of 10 weeks with a once-weekly session of 20 min and limited training adherence. This finding suggests that an excellent response in a training program with high potential for improving stepping performances relevant for avoiding falls can be achieved already with a low training frequency and short training period. Furthermore, it confirms previous research showing that the addition of this stepping exergame training component complements the effects of conventional balance training [44].

In sub-session 1, significant training effects were observed for levels with low to moderate complexity, but not for those with the highest complexity. This finding could be caused by the lower statistical power resulting from the smaller sample sizes in these more complex levels. As a result of piloting and defining an appropriate entry level of difficulty for a sample of older adults with functional limitations, excellent completion rates with no floor effects were achieved for the initial levels. However, due to the progression of task difficulty according to the individual performance of each participant, completion rates decreased throughout the levels with progression to more complex levels. Such an assessment approach with entry difficulty levels based on pilot pretests and progression of difficulty has already been successfully used for an exergame-based assessment with comparable level structure in older adults with CI [20]. This approach prevents ceiling effects for the higher functioning participants and enables the assessment of the individual upper maximum performance levels of each participant (‘testing the limits paradigm’),

In sub-session 2, participants had either to inhibit a stepping reaction when moving triangles appeared instead of squares, or to react also to moving triangles if static squares switched to triangles. As response inhibition during stepping has been identified as an independent risk factor for falls [9], these exergame tasks, in which participants had to deal with several distractors under challenging cognitive load while performing appropriately timed step movements, had been included in this study. Compared to sub-session 1, training effects in these combined stepping and response inhibition tasks were lower. This might be explained by a potential ceiling effect across the levels of sub-session 2. The task structure of these levels demanded participants to restrain reactions for inhibiting irrelevant task stimuli, which might have favored the performance of low and high-functioning participants due to the possibility of unconscious, but correct non-reactions. In line with this assumption, more participants met the level completion criteria and reached higher levels in sub-session 2. This might have led to high performances prior to and after the intervention, limiting the room of improvements and the potential impact of the intervention on the levels of sub-session 2.

As documented by the predominantly large to very large training effects on the (sub-)total SRT and game score outcomes for both sub-sessions, this study documents the effectiveness of the stepping exergame training to substantially improve the overall exergame-based stepping performance in a sample of older adults with on average moderate functional limitations and relevant comorbidities. Our results correspond to those reported in previous studies for the effects of stepping exergame interventions on the stepping performance of older adults, in which, however, stepping performance was assessed by not comprehensively validated ‘internal outcome measures’, while the sample included higher functioning older adults [14, 15].

### Sustainability of effects

Training gains decreased after cessation of the stepping exergame training, but the significant moderate to large effects observed for the stepping performance in individual levels as well as for the overall stepping performance in the sub-sessions and total session at follow-up indicated the sustainability of the motor-cognitive stepping exergame training for at least a 10-week period. Although the evaluation of long-term effects after training cessation is crucial to ensure the real benefit and practical value of exergame interventions [20], surprisingly previous RCTs have not yet evaluated the sustainability of a stepping exergame intervention on the stepping performance in older adults [14–16, 26]. To the best of our knowledge, this is the first study to report sustainable improvements in complex motor-cognitive stepping exergame tasks that address an interaction of sensory, cognitive and motor skills like that required to avoid falls in everyday life. These findings are in line with a RCT in older adults that integrated virtual stepping exercises in one of the treatment arms, in addition to strength and balance exercises, showing effects up to one year following this combined on both motor and cognitive fall-related outcomes [45, 46]. Remarkably, the sustainable improvements in our study were observed after a relatively short training period with a rather low training frequency, suggesting the high practical value of the motor-cognitive stepping exergame training to represent an effective training measure to sustainably reduce fall risk factors in older adults.

### Limitations

The study has some limitations. First, the completion rates decreased with progression to more complex levels, which limited the statistical power due to smaller sample sizes for these levels. Due to the level structure of the exergame, this pattern of completion rates was, however, expected and ensured the assessment of each participant’s individual maximal performance by progressive difficulty levels to prevent floor and ceiling effects [20, 27]. Second, the study sample comprised cognitively intact older adults with low-to-moderate functional limitations, so our findings cannot be generalized to more frail older people with CI. Third, participants were predominantly females, limiting the generalizability of our findings to males. However, we do not expect gender differences in training response to the stepping exergame training. Fourth, although we performed a highly task-specific stepping exergame training and used an assessment strategy specifically developed to document the specific intervention effects of this training, time-related effects due to the different time spent in training session by the IG and CG cannot be completely excluded. Finally, we did not include secondary, generic outcome measures such as functional or psychological status not documented effects on number of falls. Although these outcomes were not included in the study aim, the clinical relevance of training gains may therefore not be proven by the present study results.

## Conclusions

The results of this study demonstrate that a stepping exergame training can sustainably improve the performance in complex motor-cognitive stepping exergame tasks targeting the interplay between dynamic postural control, sensory functions and different cognitive subdomains that are highly relevant to initiate step responses to prevent falls in everyday life. This study is the first to document the effectiveness and sustainability of such a training program on the stepping performance in older adults using comprehensively validated, highly task-specific internal outcome measures. By effectively improving relevant cognitive, motor and sensory performances, the stepping exergame training might represent an effective and sustainable training measure to reduce the fall risk of older adults. Further studies with larger sample sizes are needed to allow for the evaluation of potential sustainable effects on fall risks and falls and the transfer of task-specific training effects to non-trained generic motor or cognitive domains.

## Supplementary information

**Additional file 1 Table S1.** Effects of the stepping exergame training on stepping reaction times [ms] (intention-to-treat analysis).

**Additional file 2 Table S2.** Effects of the stepping exergame training on game scores [%] (intention-to-treat analysis).

## Data Availability

The datasets used and/or analyzed during the current study are available from the corresponding author on reasonable request.

## Abbreviations

ANOVA: Analysis of variance
BMI: Body-Mass-Index
CI: Cognitive impairment
CG: Control group
FES-I: Falls Efficacy Scale International
IG: Intervention group
IQR: Interquartile range
MMSE: Mini-Mental State Examination
RCT: Randomized controlled trial
REGE: Geriatric rehabilitation sports club
SD: Standard deviation
SF-12: 12-item Short Form Health Survey
SPPB: Short Physical Performance Battery
SRT: Stepping reaction time
