# Supplementary material for: Effectiveness and sustainability of a motor-cognitive stepping exergame training on stepping performance in older adults: a randomized controlled trial
Source: Eur Rev Aging Phys Act. 2020 Sep 29;17:17. doi: 10.1186/s11556-020-00248-4 (PMC7525984; doi:10.1186/s11556-020-00248-4)
Supplement: Supplementary file 1 — Additional file 1 Table S1. Effects of the stepping exergame training on stepping reaction times [ms] (intention-to-treat analysis). [file 11556_2020_248_MOESM1_ESM.docx]

**Table S1.** Effects of the stepping exergame training on stepping reaction times [ms] (intention-to-treat analysis)

|  |  | T1 | T2 | T2 |  | T1 – T2 | | |  | T1 – T3 | | | |
| --- | --- | --- | --- | --- | --- | --- | --- | --- | --- | --- | --- | --- | --- |
| *Sub-session 1* | *n* | Mean (SE) | Mean (SE) | Mean (SE) |  | % change*  Mean (SE) | *P*-value^†^ | *η_p_^2^*^†^ |  | % change*  Mean (SE) | *P*-value^†^ | *η_p_^2^*^†^ | |
| Level 1 |  |  |  |  |  |  |  |  |  |  |  |  | |
| CG | 29 | 172 (15) | 154 (14) | 127 (10) |  | -3.3 (8.2) | **0.003** | 0.180 |  | -17.6 (7.6) | 0.433 | 0.019 | |
| IG | 29 | 198 (10) | 114 (12) | 127 (13) |  | -40.7 (5.5) |  |  |  | -34.0 (6.8) |  |  | |
| Level 2 |  |  |  |  |  |  |  |  |  |  |  |  | |
| CG | 24 | 197 (16) | 172 (13) | 134 (13) |  | -7.5 (6.2) | **0.003** | 0.208 |  | -24.1 (8.5) | 0.827 | 0.001 | |
| IG | 24 | 290 (24) | 140 (13) | 142 (13) |  | -45.2 (7.2) |  |  |  | -43.5 (8.1) |  |  | |
| Level 3 |  |  |  |  |  |  |  |  |  |  |  |  | |
| CG | 22 | 164 (12) | 185 (32) | 149 (25) |  | +17.0 (16.2) | 0.244 | 0.065 |  | -7.5 (12.7) | **0.034** | 0.122 | |
| IG | 21 | 244 (17) | 170 (67) | 127 (14) |  | -15.9 (46.2) |  |  |  | -44.0 (7.5) |  |  | |
| Level 4 |  |  |  |  |  |  |  |  |  |  |  |  | |
| CG | 20 | 149 (14) | 143 (11) | 150 (12) |  | +6.7 (10.5) | **0.028** | 0.134 |  | +9.6 (9.9) | **0.014** | 0.166 | |
| IG | 17 | 167 (12) | 113 (7) | 118 (7) |  | -28.9 (5.2) |  |  |  | -26.1 (5.1) |  |  | |
| Level 5 |  |  |  |  |  |  |  |  |  |  |  |  | |
| CG | 14 | 170 (17) | 132 (7) | 160 (17) |  | -10.5 (10.5) | 0.900 | 0.001 |  | -0.3 (9.8) | 0.954 | <0.001 | |
| IG | 7 | 151 (26) | 138 (21) | 153 (26) |  | +11.8 (28.7) |  |  |  | +20.5 (26.0) |  |  | |
| Sub-total score |  |  |  |  |  |  |  |  |  |  |  |  | |
| CG | 29 | 185 (14) | 162 (8) | 138 (7) |  | -7.8 (4.0) | **<0.001** | 0.357 |  | -20.4 (4.4) | **0.041** | 0.202 | |
| IG | 29 | 227 (14) | 125 (8) | 132 (8) |  | -42.2 (4.1) |  |  |  | -39.2 (3.7) |  |  | |
| *Sub-session 2* |  |  |  |  |  |  |  |  |  |  |  |  | |
| Level 6 |  |  |  |  |  |  |  |  |  |  |  |  | |
| CG | 29 | 170 (15) | 160 (28) | 125 (8) |  | +7.5 (13.0) | **0.047** | 0.070 |  | -10.7 (10.1) | 0.099 | 0.049 | |
| IG | 29 | 199 (23) | 130 (10) | 111 (8) |  | -22.3 (6.4) |  |  |  | -33.9 (5.2) |  |  | |
| Level 7 |  |  |  |  |  |  |  |  |  |  |  |  | |
| CG | 29 | 192 (22) | 149 (28) | 143 (15) |  | -11.1 (9.1) | 0.110 | 0.046 |  | -8.5 (9.2) | 0.185 | 0.032 | |
| IG | 29 | 199 (29) | 110 (10) | 119 (10) |  | -29.1 (7.5) |  |  |  | -24.3 (7.7) |  |  | |
| Level 8 |  |  |  |  |  |  |  |  |  |  |  |  | |
| CG | 28 | 168 (21) | 125 (11) | 147 (13) |  | -10.6 (9.1) | 0.486 | 0.009 |  | +8.5 (13.2) | 0.183 | 0.033 | |
| IG | 29 | 179 (18) | 128 (13) | 141 (20) |  | -23.6 (5.4) |  |  |  | -13.6 (11.2) |  |  | |
| Level 9 |  |  |  |  |  |  |  |  |  |  |  |  | |
| CG | 28 | 166 (22) | 148 (12) | 152 (18) |  | +3.5 (9.4) | 0.333 | 0.017 |  | -6.5 (9.5) | 0.783 | 0.001 | |
| IG | 29 | 195 (25) | 151 (18) | 134 (14) |  | -10.9 (8.7) |  |  |  | -14.3 (8.9) |  |  | |
| Level 10 |  |  |  |  |  |  |  |  |  |  |  |  | |
| CG | 22 | 225 (21) | 195 (16) | 161 (14) |  | -5.5 (6.7) | **0.008** | 0.166 |  | -19.5 (7.7) | 0.850 | 0.001 | |
| IG | 21 | 306 (30) | 160 (14) | 174 (16) |  | -39.0 (8.2) |  |  |  | -33.3 (8.0) |  |  | |
| Sub-total score |  |  |  |  |  |  |  |  |  |  |  |  | |
| CG | 29 | 186 (15) | 168 (25) | 146 (10) |  | -7.0 (8.3) | **0.016** | 0.101 |  | -16.2 (4.9) | 0.070 | 0.059 | |
| IG | 29 | 214 (19) | 140 (11) | 140 (11) |  | -30.4 (4.5) |  |  |  | -30.2 (4.4) |  |  | |
| *Total score (sub-session 1 & 2)* | | | | | | | | | | | | |  |
| CG | 29 | 186 (13) | 165 (15) | 142 (8) |  | -8.0 (5.2) | **<0.001** | 0.311 |  | -19.3 (4.0) | **0.019** | 0.097 |  |
| IG | 29 | 221 (15) | 132 (9) | 136 (8) |  | -38.1 (3.5) |  |  |  | -36.3 (3.5) |  |  |  |
| Data are given as pooled means (standard error, SE), % changes, *p*-values and *η_p_^2^* across five imputations. Stepping reaction times were log-transformed to satisfy the normality assumption for statistical analysis.  * calculated as follows: ((retest score – baseline score) / baseline score) × 100.  ^†^ *P*-values and effect sizes (*η_p_^2^)* are given for group effects with adjustment for baseline covariates as calculated by analysis of covariance (ANCOVA). Significant *p*-values < 0.05 are marked in bold.  T1 = baseline assessment before training, T2 = assessment after the 10-week training period, T3 = assessment 10 weeks after training cessation, CG = control group, IG = intervention group. | | | | | | | | | | | | |  |
