# Supplementary material for: Effectiveness and sustainability of a motor-cognitive stepping exergame training on stepping performance in older adults: a randomized controlled trial
Source: Eur Rev Aging Phys Act. 2020 Sep 29;17:17. doi: 10.1186/s11556-020-00248-4 (PMC7525984; doi:10.1186/s11556-020-00248-4)
Supplement: Supplementary file 2 — Additional file 2 Table S2. Effects of the stepping exergame training on game scores [%] (intention-to-treat analysis). [file 11556_2020_248_MOESM2_ESM.docx]

**Table S2.** Effects of the stepping exergame training on game scores [%] (intention-to-treat analysis)

|  |  | T1 | T2 | T2 |  | T1 – T2 | | |  | T1 – T3 | | |
| --- | --- | --- | --- | --- | --- | --- | --- | --- | --- | --- | --- | --- |
| *Sub-session 1* | *n* | Mean (SE) | Mean (SE) | Mean (SE) |  | % change*  Mean (SE) | *P*-value^†^ | *η_p_^2^*^†^ |  | % change*  Mean (SE) | *P*-value^†^ | *η_p_^2^*^†^ |
| Level 1 |  |  |  |  |  |  |  |  |  |  |  |  |
| CG | 29 | 54.9 (2.5) | 56.6 (3.2) | 61.6 (2.7) |  | +10.4 (9.6) | **0.011** | 0.125 |  | +17.2 (6.2) | 0.331 | 0.020 |
| IG | 29 | 45.9 (2.3) | 66.3 (3.1) | 62.1 (2.8) |  | +53.3 (10.9) |  |  |  | +42.9 (8.8) |  |  |
| Level 2 |  |  |  |  |  |  |  |  |  |  |  |  |
| CG | 24 | 47.9 (2.6) | 49.2 (3.8) | 58.8 (3.7) |  | +7.1 (9.8) | **0.038** | 0.165 |  | +30.3 (9.9) | 0.813 | 0.003 |
| IG | 24 | 39.0 (2.4) | 60.1 (3.6) | 58.0 (3.0) |  | +69.5 (15.1) |  |  |  | +60.1 (11.9) |  |  |
| Level 3 |  |  |  |  |  |  |  |  |  |  |  |  |
| CG | 22 | 51.6 (2.3) | 50.0 (3.5) | 56.4 (3.6) |  | -1.4 (8.4) | 0.058 | 0.146 |  | +11.3 (7.9) | 0.189 | 0.076 |
| IG | 21 | 40.0 (2.1) | 57.6 (4.1) | 58.2 (4.3) |  | +51.8 (12.1) |  |  |  | +52.6 (12.6) |  |  |
| Level 4 |  |  |  |  |  |  |  |  |  |  |  |  |
| CG | 20 | 59.0 (3.0) | 57.7 (2.5) | 58.4 (2.6) |  | +3.8 (7.7) | 0.340 | 0.027 |  | +4.6 (8.1) | 0.107 | 0.076 |
| IG | 17 | 52.3 (2.1) | 59.7 (2.9) | 62.4 (3.0) |  | +14.7 (4.5) |  |  |  | +20.1 (4.8) |  |  |
| Level 5 |  |  |  |  |  |  |  |  |  |  |  |  |
| CG | 14 | 47.2 (3.9) | 53.3 (2.5) | 47.5 (4.0) |  | +24.3 (12.5) | 0.837 | 0.003 |  | +8.3 (11.7) | 0.880 | 0.001 |
| IG | 7 | 52.0 (6.9) | 52.4 (8.2) | 49.3 (7.6) |  | +11.8 (20.6) |  |  |  | +7.4 (21.8) |  |  |
| Sub-total score |  |  |  |  |  |  |  |  |  |  |  |  |
| CG | 29 | 50.8 (1.8) | 52.5 (1.8) | 56.6 (1.9) |  | +6.2 (4.6) | **<0.001** | 0.257 |  | +13.6 (3.9) | **0.034** | 0.080 |
| IG | 29 | 44.1 (1.9) | 61.1 (2.1) | 58.6 (2.1) |  | +43.2 (6.0) |  |  |  | +37.2 (5.8) |  |  |
| *Sub-session 2* |  |  |  |  |  |  |  |  |  |  |  |  |
| Level 6 |  |  |  |  |  |  |  |  |  |  |  |  |
| CG | 29 | 52.7 (3.4) | 55.6 (4.2) | 59.2 (2.9) |  | +17.8 (12.8) | 0.299 | 0.031 |  | +29.3 (12.5) | 0.100 | 0.049 |
| IG | 29 | 51.3 (3.2) | 60.8 (4.5) | 64.8 (2.5) |  | +29.5 (11.6) |  |  |  | +38.4 (9.7) |  |  |
| Level 7 |  |  |  |  |  |  |  |  |  |  |  |  |
| CG | 29 | 56.7 (2.4) | 60.7 (2.6) | 61.0 (2.9) |  | +10.8 (5.7) | **0.009** | 0.120 |  | +12.4 (7.2) | 0.168 | 0.035 |
| IG | 29 | 51.0 (3.6) | 66.9 (2.6) | 63.6 (2.8) |  | +62.1 (22.4) |  |  |  | +56.5 (25.4) |  |  |
| Level 8 |  |  |  |  |  |  |  |  |  |  |  |  |
| CG | 28 | 52.4 (3.0) | 59.0 (2.4) | 55.0 (2.7) |  | +21.8 (8.2) | 0.359 | 0.016 |  | +15.9 (9.7) | 0.134 | 0.042 |
| IG | 29 | 49.5 (2.8) | 60.1 (2.8) | 59.3 (2.9) |  | +30.0 (8.6) |  |  |  | +31.1 (10.3) |  |  |
| Level 9 |  |  |  |  |  |  |  |  |  |  |  |  |
| CG | 28 | 57.1 (2.6) | 55.9 (2.5) | 59.3 (2.7) |  | +9.4 (13.2) | 0.322 | 0.019 |  | +16.6 (14.4) | 0.634 | 0.005 |
| IG | 29 | 50.9 (3.3) | 57.0 (3.6) | 59.1 (3.4) |  | +25.3 (10.9) |  |  |  | +36.2 (17.1) |  |  |
| Level 10 |  |  |  |  |  |  |  |  |  |  |  |  |
| CG | 22 | 36.8 (4.2) | 43.3 (3.5) | 49.9 (3.1) |  | +24.0 (11.1) | 0.071 | 0.084 |  | +55.0 (18.4) | 0.846 | 0.001 |
| IG | 21 | 27.7 (4.2) | 48.2 (4.2) | 48.2 (3.6) |  | +205.4 (63.4) |  |  |  | +217.8 (69.7) |  |  |
| Sub-total score |  |  |  |  |  |  |  |  |  |  |  |  |
| CG | 29 | 49.8 (2.3) | 54.5 (2.1) | 55.2 (2.2) |  | +17.5 (9.8) | **0.028** | 0.088 |  | +18.3 (9.0) | 0.118 | 0.044 |
| IG | 29 | 44.1 (2.6) | 58.6 (2.6) | 56.6 (2.5) |  | +40.5 (7.6) |  |  |  | +35.9 (7.5) |  |  |
| *Total score (sub-session 1 & 2)* | | | | | | | | | | | | |
| CG | 29 | 50.3 (1.8) | 53.4 (1.7) | 55.9 (1.9) |  | +8.3 (3.6) | **<0.001** | 0.236 |  | +13.1 (3.6) | **0.024** | 0.092 |
| IG | 29 | 44.1 (2.1) | 59.8 (2.2) | 57.5 (2.1) |  | +40.2 (5.9) |  |  |  | +34.3 (5.3) |  |  |
| Data are given as pooled means (standard error, SE), % changes, *p*-values and *η_p_^2^* across five imputations.  * calculated as follows: ((retest score – baseline score) / baseline score) × 100.  ^†^ *P*-values and effect sizes (*η_p_^2^)* are given for group effects with adjustment for baseline covariates as calculated by analysis of covariance (ANCOVA). Significant *p*-values < 0.05 are marked in bold.  T1 = baseline assessment before training, T2 = assessment after the 10-week training period, T3 = assessment 10 weeks after training cessation, CG = control group, IG = intervention group. Significant *p*-values < 0.05 are marked in bold. | | | | | | | | | | | | |
